# Genes of cancer-related fatigue: a scoping review

**DOI:** 10.3389/fonc.2024.1446321

**Published:** 2024-09-20

**Authors:** Yuqing Song, Xuefeng Sun, Lu Shen, Zihan Qu, Jiawei Yin, Zilin Wang, Hongshi Zhang

**Affiliations:** College of Nursing, Changchun University of Chinese Medicine, Changchun, Jilin, China

**Keywords:** cancer-related fatigue_1_, fatigue_2_, gene_3_, tumor_4_, scoping review_5_

## Abstract

**Background:**

Cancer-related fatigue (CRF) is a prevalent adverse effect experienced by cancer patients while receiving and after treatment, impacting as many as 90% of individuals. Although CRF is common, the genetic processes responsible for it and their influence on individual vulnerability are not well understood and are still being investigated.

**Objective:**

The primary objective of this scoping review is to identify and assess genes linked to the vulnerability and severity of CRF. This will help us better understand the genetic factors involved and assist in developing targeted nursing treatments in clinical settings.

**Methods:**

This review followed the PRISMA guidelines. A comprehensive search was performed in databases, such as PubMed, EMBASE, Web of Science, Cochrane Library, SinoMed, CNKI, and VIP, encompassing genetic association studies on CRF published up to February 25, 2024. The JBI Critical Appraisal Tools were used to assess the quality of observational studies.

**Results:**

This evaluation encompassed a comprehensive analysis of 14 studies that involved 3,254 patients. The results indicate strong connections between CRF and various inflammatory cytokines (IL-4, IL-6, IL-8, IL-10, IL-1β), tumor necrosis factor-alpha (TNF-α), catechol-O-methyltransferase (COMT), and circadian rhythm genes (CLOCK, PER).

**Conclusion:**

This scoping review emphasizes the significant genetic factor in CRF, with multiple genes showing distinct effects on cancer fatigue symptoms. Identifying these genes enhances our comprehension of CRF and unveils novel avenues for cancer treatment approaches. Future research should prioritize conducting cohort studies to monitor alterations in gene expression pre- and post-treatment, hence improving individualized medicinal strategies in oncology.

## 1 Introduction

The International Agency for Research on Cancer (IARC) of the World Health Organization (WHO) has recently released revised projections about the worldwide cancer burden. According to these estimates, in the year 2022, there would be around 20 million new cases of cancer diagnosed and 9.7 million deaths attributed to cancer (1). Cancer-related fatigue (CRF) is characterized as a distressing and persistent subjective feeling of physical, emotional, and cognitive tiredness or exhaustion. This fatigue is not proportional to recent activity and considerably hinders normal functioning (2). It is a frequently reported and highly distressing symptom that patients often experience when undergoing chemotherapy (3, 4). Studies have shown that more than 50% of patients have CRF during and after treatment (5). Although the exact mechanisms are not fully understood, several hypotheses have been proposed to explain the causes of CRF. These hypotheses include dysfunction in the hypothalamic-pituitary-adrenal (HPA) axis (6), the release of inflammation-related factors (7), disturbances in adenosine triphosphate metabolism (8), dysregulation of serotonin (5-HT) neurotransmission (9), changes in circadian rhythms, and vagal nerve activity (10). Furthermore, emotional responses such as stress, anxiety, and depression due to a cancer diagnosis (11), as well as issues like cancer-related insomnia and decreased sleep quality (12), have also been identified as contributing factors to CRF, leading directly to daytime fatigue.

While no specific genes have been directly linked to the onset of CRF, many studies have explored the potential role of single nucleotide polymorphisms (SNPs) in immune and inflammatory pathways. These pathways include pro-inflammatory cytokines such as IL-1β, IL-10 (13), and tumor necrosis factor-alpha (TNF-α) (14), and also IL-4 (15) and IL-8 (16). Hajj et al. showed the associations between SNPs in the dopamine receptor (DRD2) and the catechol-O-methyltransferase (COMT) gene (17). Furthermore, researchers also investigated genes closely associated with sleep quality and circadian rhythm regulation, such as CLOCK and PER, whose dysfunction may exacerbate the clinical manifestations of CRF (17).

This study aims to conduct a scoping review to deeply explore current findings on genes associated with oncogenic fatigue, collating and integrating the candidate genes and their phenotypic expressions mentioned in various studies. This study examines the role of these genes in the development of CRF by studying their biochemical pathways and evaluating their influence on fatigue at different stages of cancer. This comprehensive study aims to define the specific functions and mechanisms of different genes in cancer-related fatigue while identifying the most influential and commonly found genetic markers. This information will offer significant guidance for future clinical practice, aiding healthcare professionals in accurately identifying fatigue phenomena in cancer patients, optimizing treatment strategies, improving patient quality of life, and establishing a strong theoretical basis for developing new, targeted interventions for cancer-induced fatigue.

## 2 Materials

### 2.1 Eligibility criteria

The inclusion criteria for this study are as follows: (1) Study types: all are observational studies, including cohort studies, cross-sectional studies, and case-control studies; (2) Study population diagnosed with cancer by medical professionals; (3) Clear fatigue assessment data results reported in the literature; (4) Literature must explicitly explore the relationship between CRF and genes; (5) Study samples must be derived from human cancer patients, including blood, tumor tissue, or other biological samples indicative of gene expression; (6) The study scope ranges from database inception to February 5, 2024.

Exclusion criteria are: (1) Studies with inappropriate study designs, including reviews, conference abstracts, comments, case reports, letters, animal experiments, and other non-original research literature; (2) Studies with inconsistent themes, including those that do not explicitly mention or fail to demonstrate research on genes related to cancer-related fatigue; (3) Studies with incomplete data, lacking key data (mean ± standard deviation, odds ratio) to express the relationship between fatigue and genes.

### 2.2 Search strategy

We followed the recommendations of the Preferred Reporting Items for Systematic Reviews and Meta-Analyses (PRISMA) guidelines (18), and the protocol was registered in PROSPERO (ID: CRD42024509956). Therefore, we conducted a comprehensive search using the PubMed, EMBASE, Web of Science, Cochrane, Sinomed, CNKI, and VIP databases to retrieve all relevant articles published from the inception of the databases to February 25, 2024.

### 2.3 Selection process

All identified citations were organized and uploaded to the EndNote reference management software for screening. After removing duplicate content, two reviewers independently conducted title and abstract screening, followed by full-text review based on eligibility criteria. Any discrepancies during the study selection process were resolved by a third party.

### 2.4 Data collection and analysis

#### 2.4.1 Data extraction

Two reviewers independently extracted study details using Excel spreadsheets, and a third researcher reviewed all data entries. The following information was extracted from each eligible study, including: first author, study design, country, main content, cancer type, average age, sample size, fatigue measurement method, fatigue score, related genes, and their data.

#### 2.4.2 Quality appraisal

The methodological quality of the studies was independently assessed by two authors using the JBI Critical Appraisal Tools. Responses to the items on the checklist were categorized as “Yes,” “No,” “Unclear,” or “Not applicable,” and the quality of studies was rated as high, moderate, or low. A study was considered of high quality if all responses were “Yes.” Studies with one or two “Unclear” or “No” responses were rated as moderate quality, while those with more than two “Unclear” or “No” responses were deemed low quality (19). Any disagreements between reviewers regarding quality assessments were resolved by a third reviewer.

#### 2.4.3 Data synthesis

Despite thorough data retrieval and screening efforts aimed at integrating relevant studies for a quantitative synthesis (i.e., meta-analysis), it was not feasible to conduct one due to significant heterogeneity–such as variability in study designs, inconsistent outcome measures, or non-uniform reporting formats–and the lack of sufficient data (mean ± standard deviation, odds ratios) or the inability to convert data into a standardized format. Consequently, this scoping review will focus on a qualitative synthesis, providing detailed descriptions and discussions on the basic characteristics, methodologies, main findings, and potential bias risks of the included studies.

## 3 Results

### 3.1 Study selection

As depicted in **Supplementary Figure 1**, we conducted a comprehensive search that yielded a total of 3,130 articles, including 13 from PubMed, 1,391 from EMBASE, 1,154 from Web of Science, 49 from Cochrane, 195 from SinoMed, 207 from CNKI, and 4 from VIP. We removed 589 duplicate articles. After assessing the titles and abstracts, 2,476 articles were excluded, leaving 76 articles. Upon further full-text assessment, ultimately, 14 studies were included in the review. The review process can be seen in **Supplementary Figure 1**.

### 3.2 Study characteristics

**Supplementary Table 1** outlines the characteristics of the studies included in this review. The studies, published between 2008 and 2022, were all observational in nature, and comprised 7 cross-sectional studies, 6 cohort studies, and 1 case-control study. Geographically, 8 studies were conducted in the USA, 2 in China, 1 in the Middle East, 1 in Germany, 1 in Spain, and 1 in Australia. Regarding cancer types, 7 studies reported exclusively on breast cancer, 1 on lung cancer, 2 on head and neck cancer, 2 on colorectal cancer, and 1 on glioma, while 1 study reported on prostate cancer, breast cancer, brain tumors, and lung cancer. In terms of cancer staging, the samples included in nine studies contained the full range of stages, i.e., I\II\III\IV, two studies analyzed stage I\II only, and two studies were designed to demonstrate cancer staging. In terms of treatment modalities, surgical therapy alone was used in five studies, chemotherapy was used as a treatment in three studies, and radiotherapy alone in one study, while a combination of surgery, radiotherapy, and chemotherapy was used in three studies, and two studies did not describe treatment. Overall, the studies involved a total of 3,254 participants. Detailed characteristics of the included studies are presented in **Supplementary Table 1**.

### 3.3 Quality appraisal

Using the JBI Critical Appraisal Tools, two researchers conducted a quality assessment of all included studies, with the results presented in **Supplementary Table 2**. Among these, 10 studies were rated as high quality, 2 as moderate quality, and 1 as low quality.

### 3.4 Cancer fatigue-related genes

#### 3.4.1 Inflammatory factor

Eight studies examined the relationship between inflammatory factors and CRF, as mentioned in the referred studies (15, 20, 21, 23, 27–29, 31).

##### 3.4.1.1 Interleukin

Collado-Hidalgo et al. (31) examined the single nucleotide polymorphisms (SNPs) in the promoters of cytokine genes in a group of 33 breast cancer survivors who experienced fatigue and 14 breast cancer survivors who did not experience fatigue. The results of the multivariate logistic regression analysis showed that the IL-1β-511 and IL-6-174 polymorphisms were independently associated with fatigue status (95% CI: 0.91–16.6, 1.12–17.9; p = 0.024, p=0.021). Nevertheless, the correlation between IL6-174 and fatigue lost its significance when controlling for age and various treatment-related factors (*p >*0.05), indicating the absence of a direct association between fatigue and IL-6 levels. Consistent with these findings, Anand D et al. (15) identified only one SNP, rs4719714 of IL-6, as significantly associated with morning fatigue and another, rs1800796, with evening fatigue, although these associations did not remain significant in multivariate analysis. Contrary to these results, Xiao et al. (27) studied 46 head and neck cancer patients undergoing intensity-modulated radiation therapy (IMRT) and found a positive correlation between changes in the inflammatory markers IL-6 and C-reactive protein (CRP) and fatigue (*p* = 0.0121, *p* = 0.0224). Following a recessive model, Cameron et al. (20) showed that individuals with the low-producing CC genotype of IL-6-174SNP had reduced fatigue (95% CI: 0.10–0.75, OR=0.27, *p* = 0.012).

The presence of the C allele of IL-1β-511 was significantly related to fatigue symptoms (OR=0.33, *p* =0.027) (31). Kord M. et al. (28) further confirmed the relationship between IL1β and fatigue, with a regression analysis showing higher fatigue risk in individuals carrying the A allele (GG + GA genotype) than in the general population (95% CI: 1.336–6.226). Cielito C et al. (29) explored the relationship between SNPs in inflammatory pathways and concurrent pain, depression, and fatigue in lung cancer patients, revealing the strongest association with the IL-8 T251A variant in late-stage patients (95% CI: 1.16–3.7, OR: 2.07, *p* = 0.014). However, patients with the T/T genotype of IL-8 T251A exhibited lower chances of severe depression or pain compared to those with A/T and A/A genotypes. The study also noted a significant association between IL-10 and fatigue in females with non-small cell lung cancer (95% CI: 0.49, OR: 0.25–0.9, *p* = 0.028), though the specifics of the association were not discussed. Complementing this, Kord M et al. (28) found that the C allele of IL10rs3024496 was associated with lower fatigue levels (95% CI: 0.172–0.682, *p* = 0.02). Furthermore, Cameron et al. (20) reported significant associations of the G allele of IL-10-1082 SNP with reduced fatigue in both dominant (95% CI: 0.09–0.95, OR: 0.29, *p* = 0.041) and recessive (95% CI: 0.13–0.98, OR: 0.36, *p* =0.046) models. A single study (15) examined the relationship between IL4rs2243248 and fatigue and found a significant association with evening fatigue (95% CI: 0.120–0.762, *p* = 0.011). The study revealed that individuals with one or two G alleles had a 70% lower chance of experiencing severe evening fatigue compared to those who did not possess this gene.

##### 3.4.1.2 Tumor necrosis factor α

Four studies (15, 20, 23, 27) examined the relationship between tumor necrosis factor-alpha (TNF-α) and fatigue. Two of these studies focused on the relationship between TNF-α and fatigue in breast cancer patients; however, Cameron et al. (20) did not observe a significant association. Conversely, while investigating CRF-related SNPs and considering physical fatigue, cognitive fatigue, and emotional fatigue as subdomains of CRF, Kühl et al. (23) found that TNF-α SNP rs3093662 was associated with physical fatigue (95% CI: 1.29–4.39, OR: 2.38, *p* = 0.01), showing the strongest association during persistent fatigue stages (95% CI: 1.30–4.70, OR: 2.47, *p* = 0.006). The association of TNF-α rs1799724 was strongest at the first follow-up (*p* = 0.005), and no significant correlation was found with emotional fatigue. Moreover, Anand D et al. (15) reported that morning fatigue was associated with variations in TNF-α rs1800629 (95% CI: 0.252–0.910, *p* = 0.025) and rs3093662 (95% CI: 1.796–24.171, *p* = 0.004), whereas evening fatigue correlated with TNF-α rs2229094 variation (95% CI: 1.389–10.110, *p* = 0.009), with the C allele of TNF-α rs2229094 associated with increased risks of coronary artery disease and type 2 diabetes. Furthermore, Xiao et al. (27) found that soluble tumor necrosis factor receptor 2 (sTNFR2) significantly increased before and after IMRT treatment (*p <*0.0001).

##### 3.4.1.3 NF-κB and IRF

Two studies conducted in 2016 (27) and 2018 (21), respectively, on the fatigue levels of head and neck cancer patients are important in this respect. In addition to the inflammatory factors previously discussed, the researchers also focused on the pro-inflammatory transcription factor NF-κB and the antiviral transcription factor IRF. The results indicated that in breast cancer patients, fatigue was significantly associated with increased expression of NF-κB-related transcripts (*p* = 0.001). For head and neck cancer patients, fatigue was associated with elevated NF-κB activity (*p* = 0.001) and reduced IRF activity.

#### 3.4.2 Catechol-O-methyltransferase

Three studies (17, 25, 30) have explored the relationship between catechol-O-methyltransferase (COMT) and fatigue in breast cancer patients. Fernández et al. (30) categorized COMT into three genotypes: Val/Val, Met/Met, and Val/Met. Their findings indicated that breast cancer survivors with the Val/Met (95% CI: 5.5–6.4) or Met/Met (95% CI: 5.8–7.0) genotypes had significantly higher fatigue scores compared to those with the Val/Val genotype (*p* = 0.01), with no difference in fatigue scores between Val/Met and Met/Met genotypes. Eshragh et al. (25) found that individuals carrying the rare C allele exhibited lower severe fatigue symptoms or were less likely to experience high levels of fatigue (95% CI: 4.1–5.4, *p* = 0.026). Aline Hajj et al. (17) also investigated the relationship between COMT and fatigue in breast cancer patients, but their results showed no significant association (*p* = 0.814).

#### 3.4.3 Circadian gene

Two studies (17, 24) investigated the relationship between circadian rhythm genes and fatigue. Terri et al. (24), examined the relation between fatigue and the circadian rhythm genes ARNTL2, CLOCK, PER1, and PER2 in glioma patients; they found that SNPs in ARNTL2 (rs922270) (OR: 1.869, *p* =0.033) and PER2 (rs934945) (OR: 0.614, *p <*0.100) were associated with fatigue. Conversely, Hajj et al. (17) explored the CLOCK and PER2 genes but found no significant association with fatigue (*p* = 0.933, *p* = 0.318). Furthermore, their study included the CRY2 gene, which showed no significant relationship with fatigue (*p* = 0.564).

#### 3.4.4 Single nucleotide polymorphism

A study (22) specifically examined the impact of the SERT gene promoter on cancer-related fatigue in individuals with colorectal cancer. The findings showed that those who carried the G allele (AG+GG genotypes) at the rs25531 locus had a 1.77-fold increased risk and severity of CRF compared to those with the AA genotype (95% CI: 1.22–2.59, OR: 1.77, *p <*0.001). Nevertheless, the difference in rs956304 within the same study did not reach statistical significance (*p* = 0.084).

#### 3.4.5 Other genes or pathways

Luo et al. (26) analyzed the association between long and short variants (LL, LS, SS) of the 5-HTTLPR (serotonin transporter gene promoter polymorphism) and fatigue in colorectal cancer patients after undergoing chemotherapy. The results indicated that the individuals with the SS genotype have a significantly higher chance of experiencing fatigue for moderate to severe chemotherapy (95% CI: 1.694–39.927, OR: 8.255, *p* = 0.009). Aline Hajj et al. (17) reported that individuals with at least one C allele of the DRD2 SNP had a significantly increased fatigue incidence rate by a factor of 4.09 (OR=4.09) compared to those with the TT genotype. Eshragh et al. (25) examined the genetic polymorphism of the β2-adrenergic receptor gene (ADRB2)—ADRB2rs1042718, noting that individuals homozygous for the rare A allele at this site had an 87% reduced likelihood of belonging to a higher fatigue category (95% CI: 0.030–0.582, *p* = 0.008). Their study identified a specific site, rs6265, on the brain-derived neurotrophic factor (BDNF) gene. Individuals who carried the A allele at this site had a 50% lower chance of being in a higher fatigue category compared to those who did not have the allele (95% CI: 0.278–0.897, *p* = 0.020).

## 4 Discussion

This scoping review examined the genetic factors associated with cancer-related fatigue in 14 research comprising 3,254 participants. The investigations focused on genes that are specifically connected to fatigue in different forms of cancer, with a particular emphasis on breast cancer (as reported in seven studies). The majority of studies examined inflammatory variables (eight studies). After reviewing the 14 included papers and further summarizing and extracting the relevant elements, the present study concluded that cancer-related fatigue was associated with genes and pathways involved in the production of inflammatory factors and the circadian rhythm, as well as COMT and various SNPs. Specifically, these genes were associated with different manifestations in the fatigue levels of patients with cancer.

IL-6, a major pro-inflammatory cytokine, has been investigated for its association with cytokine activity and CRF (32). The present review observed inconsistent findings on the relationship between IL-6 levels and CRF, with two studies suggesting a relationship between IL-6 and fatigue, possibly due to IL-6 crossing the blood-brain barrier and influencing central nervous system (CNS) functions, thus exacerbating fatigue (33). In addition, genetic variants, such as the IL-6-174 polymorphism, are also known to further enhance fatigue by their effects on IL-6 synthesis and release, thus highlighting the genetic dimensions underlying an individual’s experience of CRF (34). While another study concluded that there was no association between IL-6 and fatigue, this study had a very small sample size of 47 individuals. Further studies with larger sample sizes are thus needed to assess the relationship between IL-6 and fatigue. IL-1β, a pro-inflammatory cytokine produced in different cells such as circulating monocytes and tissue macrophages (28), induces neuronal activity in the brain, resulting in subsequent fatigue (35). Research has demonstrated that IL-1βdirectly connects widespread inflammation to symptoms of weariness by impacting the control of body temperature, stimulating the hypothalamic-pituitary-adrenal axis, and causing inflammation in the brain (36). IL-8, a chemokine and a significant modulator of inflammatory reactions, exhibits increased levels in individuals suffering from chronic back pain (37), fibromyalgia (38), and chronic fatigue syndrome (39). The T251A polymorphism of the IL-8 gene is strongly linked to fatigue, pain, and depression in non-Hispanic white patients with non-small cell lung cancer (29). IL-10 has been identified as a factor that hampers the synthesis of human cytokines and has been demonstrated to impede the generation and activity of all pro-inflammatory cytokines (40). According to the research provided, patients who have the C allele of IL-10 usually do not feel significant fatigue, whereas those with the G allele tend to have lower levels of fatigue severity. Nevertheless, Kober et al. (41) discovered conflicting indications of the IL-10 pathway in breast cancer patients who exhibited various degrees of nighttime weariness while undergoing chemotherapy. This discrepancy may be attributed to distinct gene transcription processes in monocytes and dendritic cell types. The key anti-inflammatory cytokine IL-4 is being explored more and more. Anand D et al. (15) observed that the uncommon “G” allele of IL4rs2243248 is linked to elevated susceptibility to glioma and breast cancer. Kühl et al. (23) highlighted the significance of IL-4 as a crucial cytokine in breast cancer survivors due to its immunoregulatory actions and indirect impact on the tumor microenvironment, closely associated with the occurrence and progression of CRF.

TNF-α is a critical pro-inflammatory cytokine associated with various inflammatory conditions and symptoms, including fatigue. Anand et al. (15) suggested that inflammatory mediators are linked to the development of fatigue both in the morning and evening. Nevertheless, morning and evening fatigue might be considered separate but interconnected symptoms, as they exhibit dissimilar physical characteristics and genetic indicators. García-González et al. (42) offered a biological viewpoint on the mechanisms underlying cancer-related fatigue in individuals who have survived breast cancer. They found that this fatigue is linked to inflammation, dysregulation of the hypothalamic-pituitary-adrenal axis, malfunction of the autonomic nerve system, and dietary factors. This emphasizes the concept of CRF as a syndrome affected by an intricate interaction of genetic, biochemical, and environmental variables, where inflammation plays a crucial role. Furthermore, the study revealed a correlation between possessing the C allele of TNFArs2229094 and elevated susceptibility to coronary artery disease (43) and type 2 diabetes (44).

In HNC patients, fatigue is closely associated with the conserved transcriptional response to adversity (CTRA) (45), characterized by upregulated pro-inflammatory signaling and downregulated antiviral responses mediated by specific transcription factors, particularly NF-κB (46). Members of the IRF family play a crucial role as key regulators in the transcription of interferon genes, activating and augmenting the body’s defenses against tumor cells as well as pathogens, such as viruses (47). Xiao et al. (21) performed a bioinformatics analysis of gene promoter regions, demonstrating that fatigue in HNC patients was associated with changes in transcription factor activities, with NF-κB showing increased activity and IRF decreased activity in peripheral blood mononuclear cells (PBMCs). Furthermore, they observed a unique pattern in HPV-related HNC patients who exhibit a mitigated CTRA response and lower levels of fatigue compared to those with HPV-unrelated HNC. Harrington et al. (48) found that NF-κB plays a crucial role in ovarian cancer by controlling pro-inflammatory reactions and immune evasion. This leads to chemotherapy resistance, maintenance of cancer stem cells, and the spread of cancer cells to other parts of the body, known as metastasis. Additionally, NF-κB creates an immunosuppressive environment within tumor cells. Conversely, when the activity of IRF transcription factors, which are important for fighting against viruses, is decreased, it weakens the body’s ability to defend against viruses. This might worsen fatigue by reducing the body’s ability to fight against tumors (21).

The COMT enzyme plays a crucial role in the metabolism degradation of catecholamines, such as dopamine, adrenaline, and noradrenaline. The activity of the COMT enzyme affects the levels of these neurotransmitters, which play a vital role in emotional regulation (49), pain perception (50), and stress response (51). The Val158Met polymorphism in the COMT gene modulates the functionality of this enzyme. Individuals with the Met/Met genotype demonstrate lower enzyme activity than those with the Val/Val genotype. As a result, there are variations in pain sensitivity and how exhaustion is perceived. Individuals possessing the Met/Met genotype may exhibit increased pain sensitivity and, thus, a greater susceptibility to fatigue (25, 30). This is probably because the neurological system has increased activation of β2-adrenergic receptors.

Biological rhythms are a complex endogenous system that controls several biological functions, such as sleep patterns (52), metabolism (53), and hormone release (54). Fernandes R et al. (55) showed the relationship between circadian rhythm disruptions (induced by genetic polymorphisms) and the complex interplay with chronic fatigue syndrome (CFS). Circadian rhythms, which govern the natural rhythms of the sleep-wake cycle, hormonal balance, and energy metabolism, are crucial for maintaining internal homeostasis. Genes like PER2 and ARNTL2 have significant involvement in the mechanisms of the biological clock, and their variations are strongly associated with the regulation of sleep homeostasis (56), hormonal imbalances (57, 58), cancer (59), and ultimately the development of CRF. Notably, ARNTL2 is a potential oncogene in humans, which plays a crucial role in tumorigenesis and tumor immunity. However, increased expression of ARNTL2 indicates an immunosuppressive tumor microenvironment (49). Recent research (24) has demonstrated a strong link between the intensity of CRF and SNPs in the circadian rhythm genes PER2 and ARNTL2 among individuals with glioma, providing valuable insights into how circadian rhythm disruptions may exacerbate fatigue in cancer patients. Furthermore, Hajj et al. (17) explored the multifactorial nature of CRF in breast cancer patients undergoing chemotherapy, including the impact of genetic polymorphisms on fatigue levels.

Ouyang et al. (22) presented increasing evidence for the genetic susceptibility to CFS, identifying the SNP rs25531 A>G in the SLC6A4 gene as a potential marker for the susceptibility and severity of fatigue in patients with colorectal cancer. The SLC6A4 gene encodes the serotonin transporter, a crucial protein that regulates serotonin reuptake from the synaptic space (60). Ouyang proposed a mechanism for the occurrence of fatigue in cancer patients. According to this mechanism, the G allele could lead to reduced transcription of the SLC6A4 gene and decreased expression of the serotonin transporter. As a result, there is a diminished serotonin reuptake in the synaptic gap, leading to the inhibition of brain function and the onset of fatigue (22).

The 5-HTTLPR polymorphism, involving variations in the length of the gene sequence in the promoter region of the serotonin transporter gene (SERT or 5-HTT), has been extensively studied due to its association with emotional regulation (61), stress response (62), and fatigue syndrome (63). The short (S) allele variant is associated with reduced efficiency of serotonin transport, leading to changes in the availability of serotonin in the synaptic gap, which may increase susceptibility to fatigue and emotional disorders in chemotherapy patients (64). When serotonin levels are not properly regulated, it can contribute to the development of psychiatric and emotional disorders (65). A retrospective analysis of 121 colorectal cancer patients (26) showed a significant association between the SS genotype of 5-HTTLPR and an increased risk of moderate to severe fatigue associated with chemotherapy, highlighting the SS genotype as an independent risk factor. These findings support the hypothesis that the reduced serotonin transport efficiency associated with the SS genotype may lead to disruptions in emotional and energetic homeostasis, resulting in increased fatigue.

During the review and discussion of cancer-related fatigue, it was observed that certain pathological conditions were associated with changes in gene expression during and after chemotherapy. These included anemia, endocrine dysfunction, cachexia, and muscle atrophy. Various studies showed that the inflammatory cytokines IL-6 and TNF-α played crucial roles in the development of cancer-associated anemia and cachexia. Research by Bower et al. (66) confirmed that upregulation of IL-6 is not only related to anemia but also exacerbates cachexia symptoms by promoting muscle protein breakdown, aggravating fatigue. Furthermore, it was found (67) that cancer-related anemia may result from erythropoietin inhibition, facilitating the suppression of erythropoiesis by cytokines (IL-6, TNF-α). In terms of endocrine dysfunction, Lee and Prather’s research (68) highlights the role of the COMT gene in regulating the body’s stress response, especially in modulating glucocorticoid processes, which can influence the patient’s energy balance and sensation of fatigue. Furthermore, the circadian rhythm genes, CLOCK and PER, regulate endocrine functions by influencing the circadian rhythm, affecting insulin secretion and glucose metabolism, and thereby impacting energy balance and fatigue (69). Regarding the 5-HTTLPR polymorphism, studies by Hariri and Holmes (70) provide evidence that this genetic variation affects mood and sleep by regulating serotonin reuptake, which is closely linked to the severity of cancer fatigue. In terms of muscle atrophy induced by influenza A virus infection, research by Radigan et al. (71) found that IL-6 promotes muscle degradation by regulating the expression of the E3 ubiquitin ligase atrogin-1, leading to impaired muscle function and potentially increased fatigue.

## 5 Strengths and limitations

An important advantage of this study is that it is the first scoping review to investigate genes associated with CRF, providing new perspectives on treating cancer patients. Nevertheless, the study has several limitations. First, most of the research examined in this review was conducted in the United States, with fewer studies from other regions, which could create a bias. Second, the study used a qualitative synthesis method without integrating a quantitative meta-analysis. Therefore, the results lack core data and may display considerable heterogeneity. Hence, future research needs to be extensive cohort studies to describe the genetic factors associated with the development of CRF. Furthermore, conducting a more thorough investigation into their specific genetic pathways holds great potential in research, paving the way for the future implementation of this knowledge in clinical settings.

## 6 Conclusion

This scoping review provides a concise overview of recent studies investigating the genetic factors associated with CRF. The results indicate a strong correlation between fatigue and abnormal expression of genes involved in pathways related to inflammatory factors, tumor necrosis factors, catechol-O-methyltransferase, and circadian rhythm. Although associations have been identified between IL-4, IL-6, IL-8, IL-10, IL-1β, TNF-α, COMT, CLOCK, PER, and 5-HTTLPR-related genes and cancer-related fatigue, the relationship between IL-6 and cancer-related fatigue remains controversial. Given the heterogeneity of the available studies, larger prospective and functional studies are needed to clarify causal relationships. Future investigations could examine genetic loci linked to CRF using genome-wide association studies (GWAS), transcriptomics, or epigenetic analysis. This would help identify the precise mechanisms via which genes control the development and advancement of CRF, offering new targets and strategies for the focused prevention and treatment of CRF.
