# Supplementary material for: Genes of cancer-related fatigue: a scoping review
Source: Front Oncol. 2024 Sep 20;14:1446321. doi: 10.3389/fonc.2024.1446321 (PMC11449716; doi:10.3389/fonc.2024.1446321)
Supplement: Supplementary file 1 [file DataSheet1.docx]

Supplementary Material

# Supplementary Figures and Tables

## Supplementary Figures


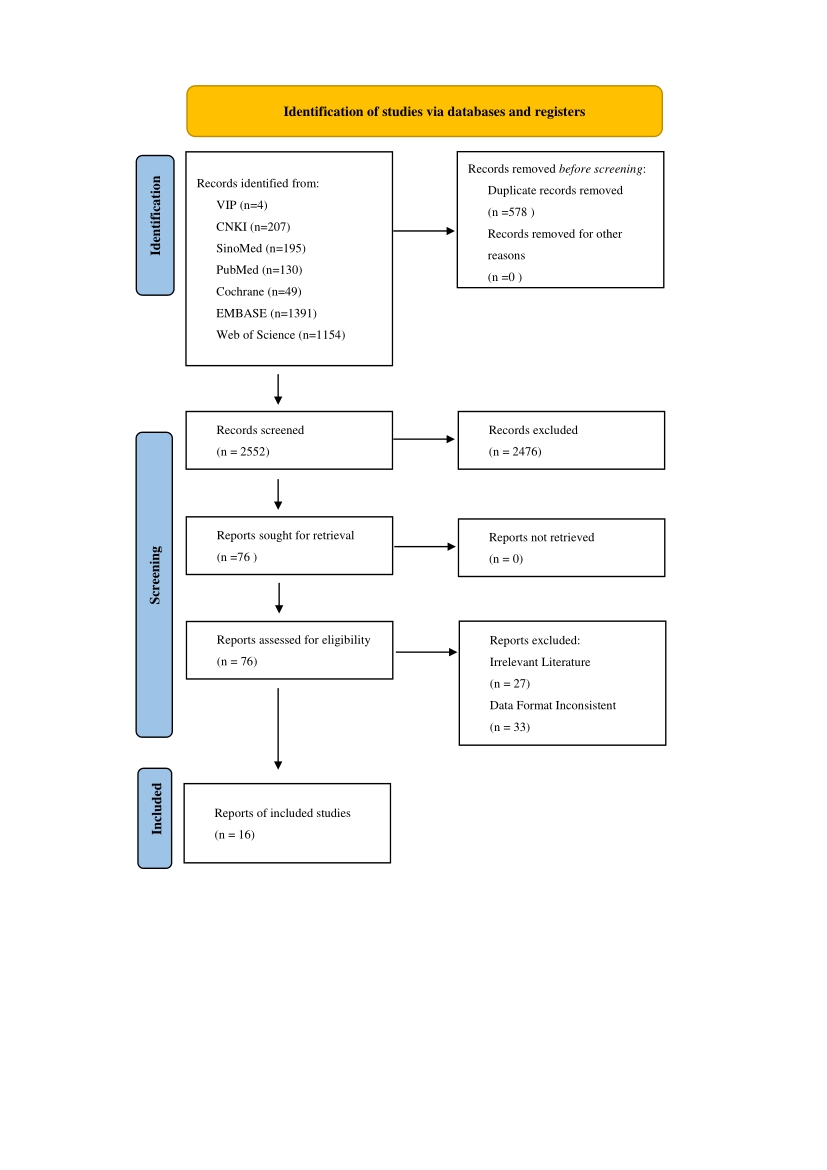


**Supplementary Figure 1.** PRISMA flow diagram

| Author/Year | Study  design | Country | Main Content | Cancer  type | Stage | Treatment | Mean age±SD | Sample size | Measurement | Gene type | Outcome  (mean±SD/OR/95%CI) | *P* value |
| --- | --- | --- | --- | --- | --- | --- | --- | --- | --- | --- | --- | --- |
| Aline Hajj 2022  (20) | Cross-  sectional | the Middle East | The aim of this study was to assess the severity of cancer-related fatigue in a group of breast cancer patients undergoing chemotherapy and explore the association between fatigue scores and sociodemographic, clinical, biological, psychiatric, and genetic factors. | breast cancer | NR | Chemo | 56.22±11.96 | 67 | EORTC QLQ-C30 | COMT rs4680 VV  COMT rs4680 VM  COMT rs4680 MM  DRD2 rs6277 CC  DRD2 rs6277 CT  DRD2 rs6277 TT  OPRM1 rs1799971 M  OPRM1 rs1799971 AG  CLOCK rs1801260 TT  CLOCK rs1801260 TC  PER2 rs934945 GG  PER2 rs934945 GA  PER2 rs934945 AA  CRY2 rs10838524 GG  CRY2 rs10838524 AG  CRY2 rs10838524 AA  ABCB1 rs1045642 CC  ABCB1 rs1045642 CT  ABCB1 rs1045642 TT | 42.59±35.8  28.77±28.77  47.92±36  59.26±31.43  42.59±31.03  34.52±31.77  43.16±31.4  39.68±36.36  42.59±33.2  43.75±30.26  37.78±32.79  48.79±30.47  55.56±38.49  44.44±31.8  44.09±32.26  35.04±34.5  36.36±26.8  34.29±34.29  38.68±32.67 | *p* =0.814  *p*=0.128  *p*=0.637  *p*=0.933  *p*=0.318  *p*=0.564  *p*=0.572 |
| B. Cameron 2021  (21) | Cohort | Australia | Cancer-related fatigue, mood disturbances, pain and cognitive disturbance are common after adjuvant cancer therapy, but vary considerably between individuals despite common disease features and treatment exposures. A genetic basis for this variability was explored in a prospective cohort. | breast cancer | I  II | Surgery：123  Chemo：136  RT:146 | 52.8±10.1 | 210 | / | s1800896 IL-10-1082  rs1800795 IL-6 -174 | Dominant model  0.29 0.09–0.95  Recessive model  0.36 0.13–0.98  0.27 0.10–0.75 | *p* =0.041  *p* =0.046  *P*=0.012 |
| Xiao 2018  (22) | Cross-  sectional | America | The aim of this study is to determine the relationship between fatigue and specific gene expression profiles associated with inflammation in human papillomavirus (HPV)-related and -unrelated HNC patients undergoing treatment. | head  and  neck cancer | ≤III  IV | RT：3  RT＋Surgery：7  RT＋Chemo：21  RT＋Chemo＋Surgery：13 | 59.05±10.39 | 44 | Multidimensional Fatigue Inventory (MFI) 20-item | NF-kB  IRF | 1.45±SE0.45  -1.25±0.48 | *p* =0.001  *p* =0.010 |
| Xi 2018  (23) | Cross-  sectional | China | To explore the correlation between the SERT gene promoter single nucleotide polymorphisms (SNPs) rs25531 and rs956304 and the cancer-related fatigue (CRF) of colon and rectal cancer, and also to analyze the correlation of the interaction of genetic and non-genetic factors. | colon  and rectal cancer | I  II  II  IV | NR | ≤60 304  ＞60 264 | 568 | Brief Fatigue Inventory (BFI-C) | SNPrs25531AG＋GG | 1.77 1.22–2.59 | *p*＜0.001 |
| T. Kühl 2018  (24) | Cohort | Germany | This study aimed to validate previously reported associations using the largest independent breast cancer sample to date and to evaluate further functional cytokine variants in relation to total CRF and all relevant CRF subdomains (physical, cognitive, and affective CRF). | breast cancer | 1  2  3  4 | Surgery | 63 | 684 | Fatigue Assessment Questionnaire | TNF α rs3093662 | Additive  2.38 1.29–4.39  Dominant  2.47 1.30–4.70 | *p* =0.01  *p* =0.006 |
| TerriS. Armstrong 2018  (25) | Cross-  sectional | America | The purpose of this study was to explore genetic variants associated with moderate to severe fatigue in patients with glioma. | glioma | I  III  IV | Surgery | None-Mild  45±13  Moderate-Severe  48±13 | 176 | the Functional Assessment of Cancer Therapy-Brain (FACT-BR) | ARNTL2 rs922270  CLOCK rs3792603  PER1 rs2253820  PER2 rs934945 | OR：1.869  1.599  0.604  0.614 | *p* = 0.033  *p* = 0 .059  *p* = 0 .089  *p* < 0.100 |
| Jasmine Eshragh 2017  (26) | Cohort | America | In patients with breast cancer, variations in neurotransmitter genes between Lower and Higher Fatigue latent classes and between the Higher and Lower Energy latent classes were evaluated. | breast cancer | 0  I  II  III | Surgery | Lower Fatigue  57.8±11.9  Higher Fatigue  53.1±11 | 397 | Lee Fatigue Scale (LFS) | ARDB2rs1042718  BDNFrs6265  COMTrs9332377  CYP3A4rs4646437  GCH1rs3783642 | 0.030-0.582  0.278-0.897  0.256-0.919  0.253-0.914  0.260-0.859 | *p*=0.008  *p*=0.020  *p*=0.026  *p*=0.025  *p*=0.014 |
| Luo 2017  (27) | Cross-  sectional | China | Analyze the association between susceptibility to chemotherapy-related fatigue in colorectal cancer patients and polymorphisms in the promoter region of the serotonin transporter gene (5-HTTLPR), specifically focusing on the long (LL) and short (SS) variants, as well as the heterozygous (LS) genotype. | colon and rectal cancer | II  III  IV | Chemo | / | 121 | Brief Fatigue Inventory (BFI-C) | 5-HTTLPR | LS  3.580 0.731-17.541  SS  8.255 1.694-39.927 | *p* =0.116  *p* =0.009 |
| Xiao 2016  (28) | Cohort | America | Patients with head and neck cancer (HNC) receiving intensity-modulated radiation therapy (IMRT) have particularly high rates of fatigue, and pre- and post-radiotherapy fatigue are prognostic factors for pathologic tumor responses and poor survival. | Head and neck cancer | ≤III  IV | RT：1  RT＋Surgery：7  RT＋Chemo：34  RT＋Chemo＋Surgery：4 | 57.76 ±10.44 | 46 | Multidimensional Fatigue Inventory (MFI) 20-item | IL6  sTNFR2  CRP | 8.04±8.67  4.49±2.37  9.59±15.44 | *p*=0.0013  *p*＜0.0001  *p*=0.6590 |
| Kord M. Kober 2016  (29) | Cohort | America | To identify subgroups (i.e., latent classes) of women with distinct fatigue and energy trajectories; evaluate for differences in phenotypic characteristics between the latent classes for fatigue and energy; and evaluate for associations between polymorphisms in genes for pro- and anti-inflammatory cytokines, their receptors, and their transcriptional regulators and latent class membership. | breast cancer | 0  I  II  III  IV | Surgery | Lower Fatigue 57.8  Higher Fatigue 53.1 | 516 | Karnofsky Performance Status (KPS) scale | IL1Brs16944  IL10rs3024496 | 1.336-6.226  0.172-0.682 | *p*=0.08  *p*=0.02 |
| Anand Dhruva 2015  (30) | Cross-  sectional | America | The purpose of this study was to evaluate for differences in variations in pro- and antiinflammatory cytokine genes between participants who were classified as having low and high levels of morning and evening fatigue and to evaluate for differences in phenotypic characteristics between these two groups. | breast, prostate, lung,  or brain cancer | NR | RT | 61.5 | 252 | Lee Fatigue Scale (LFS), | TNFArs1800629  TNFArs3093662  IL4rs2243248  TNFArs2229094 | 0.252-0.910  1.796-24.171  0.120-0.762  1.389-10.110 | *p*=0.025  *p*=0.004  *p*=0.011  *p*=0.009 |
| Cielito C. Reyes-Gibby 2013  (31) | Cross-  sectional | America | We applied novel statistical methods to assess whether variants of 37 inflammation genes may serve as biologic markers of risk for severe pai》 | non-small cell lung cancer | I-IIIA  IIIB-IV | NR | 61±12 | 97 | 12-Item Short-Form Health Survey (SF-12) | IL-8-T251 A  IL-10 | 2.07 1.16-3.70  0.49 0.25-0.92 | *p*=0.014  *p*=0.028 |
| Fernandez 2012  (32) | Cross-  sectional | Spain | Our aim was to examine the influence of catechol-O-methyltransferase (COMT) Val158Met genotypes on cancer-related fatigue, postmastectomy pain, and pressure pain hypersensitivity in breast cancer survivors.. | breast cancer | I-IIIA | Surgery | 25-65 | 128 | Piper fatigue scale (PFS) | COMT Val/Val  COMT Val/Met  COMT Met/Met | 4.8±1.7（4.1-5.4）  5.9±1.7（5.5-6.4）  6.4±1.6（5.8-7.0） | *p*＜0.001 |
| Alicia 2008  (33) | Case-control | America | We examined single nucleotide polymorphisms (SNPs) in the promoters of cytokine genes as genetic risk factors for cytokine-related fatigue in 33 fatigued and 14 non-fatigued breast cancer survivors, focusing on promoter sequence polymorphisms in IL1B and IL6 associated with differential expression of proinflammatory cytokines. | breast cancer | 0  I  II | Chemo | Fatigued  54.1 ± 8.3  Non-fatigued  61.1 ± 8.5 | 47 | vitality subscale of the SF-36 | IL 1B −511（C/T）  IL 6 −174（G/C） | 0.91-16.6  1.12-17.9 | *p*=0.007  *p*=0.027 |

**Supplementary Table 1.**Characteristics of the included studies.

| Author/Year | Year | Study design | Mean age | Sample size | JBI quality assessment outcome |
| --- | --- | --- | --- | --- | --- |
| Aline Haj(20) | 2022 | Cross- sectional | 56.22±11.96 | 67 | High |
| B. Cameron(21) | 2021 | Cohort | 52.8±10.1 | 111 | Low |
| Canhua Xiao(22) | 2018 | Cohort | 59.05±10.39 | 44 | Medium |
| Xi Ouyang(23) | 2018 | Cross- sectional | N/A | 568 | High |
| T. Kühl(24) | 2018 | Cohort | 63 | 684 | High |
| Terri S. Armstrong(25) | 2018 | Cross- sectional | Mild group  45±13  Severe group  48±13 | 176 | Medium |
| Jasmine Eshragh(26) | 2017 | Cohort | Low fatigue  57.7±11.9  High fatigue  53.1±11 | 397 | Medium |
| Luo(27) | 2017 | Cross- sectional | N/A | 121 | High |
| Canhua Xiao(28) | 2016 | Cohort | 57.76 ±10.44 | 46 | High |
| Kord M. Kober(29) | 2016 | Cohort | 57.8/53.1 | 516 | High |
| Anand Dhruva(30) | 2015 | Cross- sectional | 61.5 | 252 | High |
| Cielito C. Reyes-Gibby | 2013 | Cross- sectional | 61±12 | 97 | High |
| Ce´sar(31)  Ferna´ndez-de-las-Pen˜as(32) | 2012 | Cross- sectional | 25-65 | 128 | High |
| Alicia Collado-Hidalgo(33) | 2008 | Cohort | 54.1 ± 8.3 | 47 | High |

**Supplementary Table 2.** Quality Assessment.
